# Diagnostic thinking and information used in clinical decision-making: a qualitative study of expert and student dental clinicians

**DOI:** 10.1186/1472-6831-10-11

**Published:** 2010-05-13

**Authors:** Gerardo Maupomé, Stuart Schrader, Saurabh Mannan, Lawrence Garetto, Hafsteinn Eggertsson

**Affiliations:** 1 Department of Preventive and Community Dentistry, Indiana University School of Dentistry, Indianapolis, Indiana, USA; 2 The Regenstrief Institute, Inc., Indianapolis, Indiana, USA; 3 Department of Oral Biology, Indiana University School of Dentistry, Indianapolis, Indiana, USA; 4 Robert Schattner Center, School of Dental Medicine, University of Pennsylvania, Philadelphia, Pennsylvania, USA; 5 Office of Dental Education, Indiana University School of Dentistry, Indianapolis, Indiana, USA

## Abstract

**Background:**

It is uncertain whether the range and frequency of Diagnostic Thinking Processes (DTP) and pieces of information (concepts) involved in dental restorative treatment planning are different between students and expert clinicians.

**Methods:**

We video-recorded dental visits with one standardized patient. Clinicians were subsequently interviewed and their cognitive strategies explored using guide questions; interviews were also recorded. Both visit and interview were content-analyzed, following the Gale and Marsden model for clinical decision-making. Limited tests used to contrast data were t, χ^2^, and Fisher's. Scott's π was used to determine inter-coder reliability.

**Results:**

Fifteen dentists and 17 senior dental students participated in visits lasting 32.0 minutes (± 12.9) among experts, and 29.9 ± 7.1 among students; contact time with patient was 26.4 ± 13.9 minutes (experts), and 22.2 ± 7.5 (students). The time elapsed between the first and the last instances of the clinician looking in the mouth was similar between experts and students. Ninety eight types of pieces of information were used in combinations with 12 DTPs. The main differences found in DTP utilization had dentists conducting diagnostic interpretations of findings with sufficient certainty to be considered definitive twice as often as students. Students resorted more often to more general or clarifying enquiry in their search for information than dentists.

**Conclusions:**

Differences in diagnostic strategies and concepts existed within clearly delimited types of cognitive processes; such processes were largely compatible with the analytic and (in particular) non-analytic approaches to clinical decision-making identified in the medical field. Because we were focused on a clinical presentation primarily made up of non-emergency treatment needs, use of other DTPs and concepts might occur when clinicians evaluate emergency treatment needs, complex rehabilitative cases, and/or medically compromised patients.

## Background

Since seminal work was carried out decades ago in Medicine [1-3], few investigations have addressed dental clinical decision-making [4-11] from the perspective of how the individual clinician uses diagnostic thinking, and incorporates concepts and strategies in his/her clinical decisions. This state of affairs is unfortunate, as decisions in dental practice could be rendered more stable and reproducible [12-14]. Many statements on how to attain a superior level of performance seem to be limited to general recommendations [15-17]. Researchers and various people involved in dental academia have looked at the challenges from perspectives rooted in paradigms championing clinical performance and pedagogical excellence. Because some of these descriptions have stressed what clinicians should be doing to achieve a desirable quality of performance [18], or attempted to examine and/or replicate the management of clinical information using algorithms [19,20], or tried to interpret the actions of clinicians when involved in diagnostic/management tasks [21], we must conclude that multiple levels of interpretation of clinical decision-making are feasible. The challenge for dental education is to understand how clinical decision-making can be characterized and purposefully improved while clearly laying out the assumptions within an interpretation framework.

Clinical decision-making is a complex phenomenon which presents the task of solving an "unknown problem" [22], a problem "ill-defined" [23]. Models have been created to facilitate the study of its complexities. Models can be paramorphic (symbolic representations of the process) or isomorphic (accurate descriptions of the process) [24]. Most models reported under the heading of 'decision-making' are paramorphic. They are analogues of the components and sequence of thoughts that make up the process. Conversely, isomorphic models are concerned with describing the actual components and their interactions. Paramorphic models of decision-making have often been used by designing statistical models [25-27]; decision analysis, decision theory and decision-tree models [28-34], or set theory models [27,35]. These models, however, are idealized diagrammatic representations of human strategies, which makes them somewhat detached from actual cognitive handling of decision-making information.

Isomorphic models have gained acceptance over time. Early work by Elstein et al. [3] hinged upon observations of clinicians at work, and led to proposing a model of medical enquiry divided into cue acquisition, hypothesis generation, cue interpretation, and hypothesis evaluation. Similar approaches continued this line of enquiry, combining observations by psychologists with the clinician's own statements after the clinical encounter [1,2]. The question of 'how' clinicians make sense of chunks of diagnostic information or how these diagnostically meaningful pieces of information are acquired, has been open to differing interpretations for some time [11,36,37]. The debate has often centered on how readily the clinician can assemble pieces of information into a clinical picture: that cognitive pathways cross-reference clinical features against pieces of information stored in memory, or that clinically meaningful 'pictures' are instantly recognized [37].

Our own past research has attempted to characterize diagnostic thinking in restorative dentistry [9,10]. We purported to study the strategies and the pieces of information that prompt utilization of specific memory structures leading to clinical interpretation, following the Gale and Marsden model [38]. The effort was grounded on a cognitive psychology approach whereby clinicians examined introspectively their clinical performance during a simulated clinical visit.

In the present manuscript, we build on our prior research and expand the sophistication of our interpretation of the clinical decision-making processes in restorative dentistry (adopting an isomorphic developmental perspective) by observing both expert clinicians and competent senior dental students. Although Crespo et al. [11] found distinctions between beginning, competent, and expert clinicians, their findings are limited because the method used involved a hypothetical paper case and not a "live" simulation. Its focus was more on the medical complexities posed by a poorly-controlled diabetic patient scenario than the appraisal of restorative needs. We have developed a standardized patient model to enhance the fidelity and realism of the clinical environment instead of using a 'paper' patient appraised under simulated clinical conditions [9,11]. The qualitative research questions of the present study are: Does the range and frequency of cognitive processes involved in diagnosis and treatment planning differ between senior student and expert clinicians? And, what are the different pieces of cognitive information used by senior student and expert clinicians while making restorative decisions?

## Methods

The study design followed ethical guidelines at Indiana University (IRB 0606-57) to protect all participants' individual rights (standardized patient and clinicians).

### Gale and Marsden (GM) model: A cognitive psychology theoretical framework

**Figure 1:**
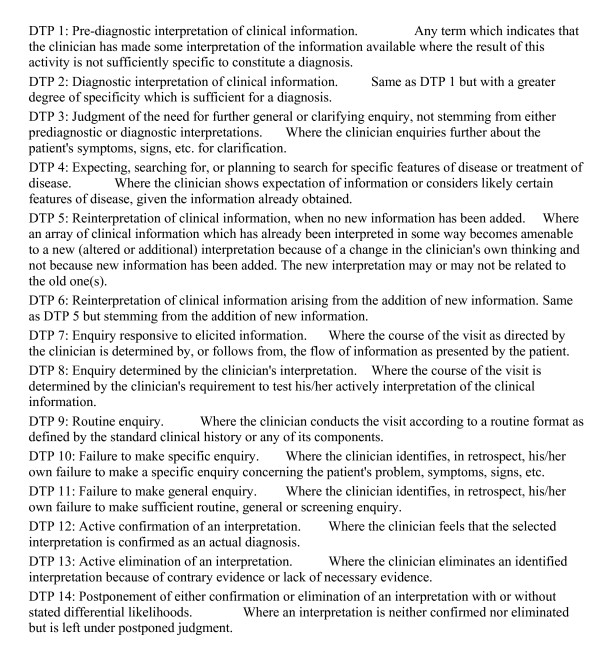
**Inventory of Diagnostic Thinking Processes**. Gale and Marsden Model of Clinical Decision-Making.

Gale and Marsden [38] described clinical decision-making by identifying cognitive processes that occurred as the clinician moves through the resolution of a clinical problem. These are called Diagnostic Thinking Processes (DTP). Figure 1 defines the DTPs, as later used in this study. Gale and Marsden [38] described their model as a perception of a diagnostic challenge, which is dependent upon the way knowledge is structured in memory. Both knowledge and its structure vary between persons. The link between knowledge structure and a clinical situation is formed by significant features within pieces of information, called 'forceful features' [39]. They are not a property of information but are derived by the clinician from experience and are part of the memory structure. Others have described pathways that are functionally similar [40]. While clinicians interpreted initial pieces of information based on how well defined a problem was before another DTP could be applied [41], there were no significant differences in the range of DTP available to experienced or student clinicians [42]. Gale and Marsden interpreted this finding as differences in expertise being mainly differences in the content and structure of memory, but not of the thought processes.

### Standardizing the patient case

#### Developing a Standardized Patient Case Scenario

The goal was to find an "everyday" type of dental patient, who could be of either sex ranging in age from 25 to 60 years old and:

• who had at least 20 teeth, with some carious lesions but no major prosthetic or endodontic needs,

• with existing restorative needs limited to class I, II, or III amalgam and composite fillings,

• with no more than 3-6 units of crown and bridge work,

• with a minor periodontal pocket profile in the 2-4 mm range and with gingival recession generally not exceeding 2 mm,

• with no urgent dental treatment needs,

• not medically compromised or treated for head or neck cancer,

• not wearing or needing removable prostheses, or having more than one dental implant,

• not having diagnosable TMD problems, exhibiting significant dental phobias, or needing pre-medication before dental appointments, and

• who did not have severe fluorosis/erosion/wear; severe malocclusion, cervical abrasions, soft tissue disorders, or obvious signs of dental/craniofacial trauma.

#### Recruiting a Standardized Patient

Initial screening involved subjects invited from a pool of simulated patients. An oral soft tissue examination was conducted. Their teeth were thoroughly examined using the International Caries Detection & Assessment System (ICDAS) criteria [43]. (ICDAS scores tooth status for presence of active caries as well as lesion severity.) One subject was selected and a panoramic film x-ray taken, supplemented by a radiographic series. Full periodontal charting was conducted, and study models were fabricated. All information was entered in AxiUm (Exan Enterprise, Las Vegas, NV), an electronic dental record system. Additionally, a dental, medical, and psychosocial history was assembled. The standardized patient was a white female who had not seen a dentist in 2-3 years during college. She had started a new job with dental insurance benefits. She had restorations with marginal discrepancies, and there was a missing cast restoration in a lower second molar that had become loose three months prior to recruitment; the patient had an (asymptomatic) fractured lingual cusp on that tooth, dressed with an over-the-counter temporary filling. Third molars had occlusal, radiographically detectable asymptomatic carious lesions.

Intra-oral images were collected of each tooth using a Suni USB digital intraoral wand type video camera (SUNI Medical Imaging, San Jose, CA): 1280 × 960 pixel pictures in TIFF format. Teeth were dried prior to imaging. After being captured, each picture was reviewed on computer monitor, and once it was deemed to be of an appropriate quality and resolution, it was saved to a data file and subsequently serialized in a MS Office PowerPoint^® ^presentation.

### Dental visit procedures and research interview

Competent fourth year dental students and dentists were compensated $100 U.S. and 3 hours continuing education credits for participating in this study, respectively. Using the standardized patient in a regular dental setting, each clinician was supplied with a description of how the patient contact would unfold, the components available in the dental visit, and the sequence of the (i) dental visit and (ii) interview exercises. Conventional video and audio-recording equipment was used to record the dental visit (TAPE 1) until terminated by the clinician. A researcher kept track of the interaction between clinician and patient, noting potential decision-making events. The clinician and researcher moved to a separate office after the clinician indicated that s/he was satisfied with the information, and a treatment plan was sketched. TAPE 1 of the dental visit was then replayed for the researcher and the clinician. The researcher interviewed the clinician while s/he had available a full series of radiographs, complete medical and dental history, and intra-oral images of teeth and mouth. A second video/audio recording was completed (TAPE 2) as the clinician recalled his/her thoughts with the researcher, to gather an account of the strategies pursued and the relevant pieces of information (concepts) used in the dental consultation.

### Content-analysis of the dental visit and research interview phases

Content analysis [44,45] was conducted by a researcher with prior training in qualitative methods and a dental background, using the discourse on TAPES 1 and 2 (visit and interview phases). Content analysis was used to link conversational and nonverbal cues within and between TAPES 1 and 2 for (i) the identification of a DTP, either by recognizing the semantics present or because the DTP's occurrence could be inferred from interview participants actions and interactions [9]. We also developed (ii) categories of concepts relevant to DTP occurrences. Categorization of concepts involved an open-ended constructivist process in which initial categories were revised so as to compare across data and to literature; namely, the categories identified in past research efforts. Then, concepts were reviewed for their repetition or frequency of use, recurrence of the same meaning with different idioms, or forcefulness. Each concept was added to a category already open, or in a new category if the coding process so required.

### Statistical Analyses

This is an inherently qualitative study. We followed standard recommendations to systematically quantify findings. The coded DTP and concept data were entered into SPSS^® ^version 15. Data were analyzed using Student's t test for differences between means, and either χ2 test for associations or Fisher's exact test for percentages. As the research was a non-experimental design, we did not attempt to adjust the α level. Our approach at data analysis was not 'mapping' or time-sequencing of actions or perceived strategies [11] but rather the frequency of use of specified cognitive processes.

Content-analysis reliability was evaluated by establishing the stability (intra-coder variability) of DTP coding [46] in four randomly selected interviews which were then blindly re-coded. The analysis was conducted using a content-analysis coefficient (Scott's π) that corrects expected chance agreement [47]. Stability coefficients for DTP coding ranged from 0.63 to 0.71. This range is considered appropriate since Scott's π is a very conservative assessment.

## Results

### Dental visit characteristics and basic results

A total of 32 clinicians participated in the dental visit and the research interview phases of the study, with 15 dentists (33% female) and 17 senior dental students (47% female) (p = 0.42). The overall visit intervals lasted a mean of 32.0 minutes (standard deviation 12.9) among experts, and 29.9 (± 7.1) among students (p = 0.57). The length of time between when clinicians first began reviewing patient information and when they requested having the patient brought into the operatory was on average 5.6 minutes (± 4.9) among experts, and 7.6 (± 3.7) among students (p = 0.20). The average face-to-face dental visit time spent with the standardized patient was 26.4 minutes (± 13.9) for experts, and 22.2 (± 7.5) for students (p = 0.25). On average 10.8 minutes (± 6.4) elapsed for experts between the first instance of looking into the mouth of the standardized patient and the last instance; these intervals were not statistically significantly different between experts (10.8 min ± 6.4) and students (13.2 ± 5.1), however, substantial variations in time did exist across all clinicians.

Fifteen dentists evaluated the radiographs before seeing the patient (as did all students).

### Use of DTPs and concepts by clinicians

Table 1 presents the frequency of utilization of overall DTP per interviewee, across the two groups of clinicians (experts and students), and by DTP. (See Figure 1 for DTP inventory and Additional file 1 for verbatim quotes of DTP examples.) A total of 627 DTP were used by the 32 clinicians, with an average of 19.7 DTPs used by students and 19.5 by dentists (p = 0.92; overall range; 9-27). All clinicians utilized all types of DTPs except DTP10 and DTP11 (*'Failures to make specific or general inquiries'*), which were not used by students or dentists. The differences in overall utilization across DTPs were considerable, with DTP3 (*'Judgment of the need for further general or clarifying enquiry'*) being used 227 times versus DTP13 being used only 2 times (*'Active elimination of an interpretation'*) (see Table 2).

**Table 1:** Diagnostic Thinking Process frequency distribution across student and expert clinicians

*Student No.*	1	2	3	4	5	6	7	8	9	10	11	12	13	14	*Total*
***1***	1	3	8	2	0	1	0	2	4	0	0	3	1	0	25
***2***	0	0	9	4	0	0	3	2	4	0	0	1	0	0	23
***3***	2	1	8	2	1	2	0	3	2	0	0	0	0	0	21
***4***	0	0	6	2	0	0	2	1	3	0	0	0	0	0	14
***5***	0	1	6	1	0	0	0	1	1	0	0	0	0	0	10
***6***	1	0	8	1	1	0	0	4	4	0	0	1	0	0	20
***7***	2	1	9	5	0	0	1	1	3	0	0	2	0	1	25
***8***	1	0	10	1	0	1	0	1	2	0	0	3	0	0	19
***9***	0	2	3	1	0	0	2	1	1	0	0	0	0	0	10
***10***	0	1	8	1	0	1	2	1	4	0	0	2	0	1	21
***11***	2	2	11	0	0	0	1	1	4	0	0	0	0	0	21
***12***	1	0	12	3	0	1	2	2	3	0	0	2	0	0	26
***13***	2	0	10	1	0	0	0	2	2	0	0	0	0	0	17
***14***	1	2	10	2	0	0	0	1	3	0	0	3	0	0	22
***15***	0	1	7	2	0	1	3	0	4	0	0	1	0	0	19
***16***	3	1	7	2	0	0	2	1	3	0	0	3	0	0	22
***17***	2	1	9	3	0	0	1	2	2	0	0	0	0	0	20

***Overall Students:***	1,1	0,9	8,3	1,9	0,1	0,4	1,1	1,5	2,9	0,0	0,0	1,2	0,1	0,1	19,7


***Dentist No.***	**1**	**2**	**3**	**4**	**5**	**6**	**7**	**8**	**9**	**10**	**11**	**12**	**13**	**14**	***Total***

***1***	1	7	2	5	1	1	1	1	4	0	0	1	0	2	26
***2***	1	0	3	1	0	0	0	1	2	0	0	1	0	0	9
***3***	0	0	3	4	0	0	0	4	5	0	0	2	0	0	18
***4***	0	0	5	0	0	0	0	1	2	0	0	1	0	0	9
***5***	0	0	5	0	0	0	0	1	2	0	0	1	0	0	9
***6***	0	3	1	1	1	2	0	0	1	0	0	0	0	0	9
***7***	2	3	6	4	0	2	2	2	4	0	0	0	1	0	26
***8***	0	3	9	4	1	0	2	1	2	0	0	0	0	1	23
***9***	0	0	4	3	0	0	1	1	2	0	0	1	0	1	13
***10***	2	4	7	3	0	1	1	2	5	0	0	1	0	1	27
***11***	1	2	4	1	0	1	1	2	6	0	0	2	0	0	20
***12***	2	2	7	2	0	0	4	2	4	0	0	0	0	0	23
***13***	0	2	6	2	0	0	1	4	7	0	0	2	0	0	24
***14***	2	2	11	3	0	1	3	1	7	0	0	2	0	0	32
***15***	1	4	13	1	0	0	0	0	3	0	0	2	0	0	24

***Overall Dentists:***	0,8	2,1	5,7	2,3	0,2	0,5	1,1	1,5	3,7	0,0	0,0	1,1	0,1	0,3	19,5

***TOTAL***	30	48	227	67	5	15	35	49	105	0	0	37	2	7	627

**Table 2:** Diagnostic Thinking Processes used by clinicians along four quarters of visit time intervals

*STUDENTS*	1	2	3	4	5	6	7	8	9	10	11	12	13	14	*Total*
***1st quarter***	0	0	17	5	0	2	0	1	18	0	0	0	0	0	43
***2nd quarter***	3	1	28	10	1	2	6	4	25	0	0	6	0	1	87
***3rd quarter***	7	9	61	8	0	1	10	14	5	0	0	6	1	0	122
***4th quarter***	8	6	35	10	1	2	3	7	1	0	0	9	0	1	83

***Overall Students***	18	16	141	33	2	7	19	26	49	0	0	21	1	2	335

***DENTISTS***	**1**	**2**	**3**	**4**	**5**	**6**	**7**	**8**	**9**	**10**	**11**	**12**	**13**	**14**	***Total***

***1st quarter***	0	0	5	4	0	0	3	1	8	0	0	0	0	0	21
***2nd quarter***	3	4	11	13	0	2	6	6	21	0	0	0	0	0	66
***3rd quarter***	6	15	37	8	0	4	6	10	15	0	0	2	0	1	104
***4th quarter***	3	13	33	9	3	2	1	6	12	0	0	14	1	4	101

***Overall Dentists***	12	32	86	34	3	8	16	23	56	0	0	16	1	5	292

***Total***	30	48	227	67	5	15	35	49	105	0	0	37	2	7	627

Conversely, differences for each one of the DTPs used by students or by dentists were relatively small. Only two DTPs were found to be statistically differently between dentists and students: DTP2 ('*Diagnostic interpretation of clinical information'*) had a lower mean use of 0.9 instances among students versus 2.1 among dentists (p = 0.0004), and DTP3 (*'Judgment of the need for further general or clarifying enquiry'*) had a higher average of 8.3 instances among students versus 5.7 among dentists (p = 0.0001). Although statistically not significantly different, DTP9 ('*Routine enquiry*') was found to be evident on average 2.9 times for students versus 3.7 for dentists. Please note that the use of DTPs described above is outlined for the entire length of the dental visit.

We wanted to ascertain the sequencing of DTP and concept uses: we made these distinctions explicit by breaking down the overall visit time for each clinician into four segments of equal length, thereby suggesting a first contact and initial gathering of information in the first 25% of the visit time, and closure and presentation of treatment plan in the last 25% of the visit time. Table 2 presents the use of DTPs in the first, second, third, and final 25% segments of the visit time across students and dentists. The frequency of DTPs increased as the visit developed from the first quarter to the third, and then subsided as the clinician wrapped up the visit in the final quarter. Overall use of DTPs was somewhat similar across students and dentists (335 vs. 292). Statistically significant differences were found among students and dentists for three DTPs: (1) DTP2 in the second quarter (students, 32.9% vs. dentists, 16.7%; p = 0.003); (2) DTP3 in the third quarter (students, 50.0% vs. dentists, 35.6%; p = 0.029) (with DTP2 approaching significance, 7.9% students vs. 14.4% dentists, p = 0.087); and (3) DTP9 in the final quarter (students, 1.2% vs. dentists, 13.5%; p = 0.005). For DTPs 7 (*'Enquiry responsive to elicited information'*) and 12 (*'Routine enquiry'*) students were seemingly (although not statistically significantly) more prone to use these diagnostic strategies later in the visit, compared with dentists.

We have described above the strategies employed by clinicians; we will now describe their use of pieces of information. There were 15 categories of 98 concepts (Table 3) used by clinicians during the dental visits and research interviews (the detailed list of concepts is in Additional file 2). Table 3 also presents the use across time quarters during the visit by student versus expert clinicians. Discrete pieces of information concepts within the 15 categories were used in association with DTPs. For example, "*I think there is some incipient decay on this occlusal surface*" would be DTP1 used jointly with concept '*undefined lesion (possible presence in situ)*'. Note that the latter is different from concepts '*undefined lesion (actual presence in situ)*', '*residual lesion (possible presence in situ)*', or '*recurrent lesion (possible presence in situ)*'. A total of 18 concepts were in the category 112 called "*Dental Caries*", which constituted one of the arrays of concepts used.

**Table 3:** Overall use of concepts in the visit and in each quarter of visit time intervals

*Family of Concepts*	Entire consultation	1st quarter	2nd quarter	3rd quarter	4th quarter
***101. HEALTH STATUS, OVERALL***					
***All clinicians***	5,83%	18,5%	10,5%	1,96%	0,9%
***Dentists***	5,82%	22,4%	9,7%	3,4%	0,9%
***Students***	5,84%	16,5%	11,6%	0,9%	0,9%
***102. PERSONAL HABITS/FEATURES***					
***All clinicians***	2,3%	7,51%	3,46%	0,89%	0,9%
***Dentists***	1,89%	8,6%	2,3%	0.00%	1,8%
***Students***	2,59%	7.00%	4,3%	1,5%	0.00%
***103. PHYSICAL STATUS, OVERALL***					
***All clinicians***	0,19%	0,58%	0,46%	0.00%	0.00%
***Dentists***	0,15%	0.00%	0,6%	0.00%	0.00%
***Students***	0,22%	0,9%	0,4%	0.00%	0.00%
***104. ATTENDANCE (to denatl care)***					
***All clinicians***	5,27%	9,83%	8,31%	4,09%	2,03%
***Dentists***	7,28%	15,5%	14,9%	5,5%	0,9%
***Students***	3,78%	7.00%	3,9%	3,1%	3,1%
***105. COMPLAINT/SIGN/SYMPTOM***					
***All clinicians***	8,62%	8,09%	13,16%	8,19%	4,95%
***Dentists***	9,17%	6,9%	13,7%	9,8%	5,5%
***Students***	8,22%	8,7%	12,8%	7.00%	4,4%
***106. ORAL HYGIENE (actions)***					
***All clinicians***	3,1%	1,16%	2,31%	3,38%	4,28%
***Dentists***	3,06%	0.00%	2,3%	2,6%	5.00%
***Students***	3,14%	1,7%	2,3%	4.00%	3,6%
***107. ORAL HYGIENE (appearance)***					
***All clinicians***	2,05%	1,16%	1,62%	1,78%	3,15%
***Dentists***	3,06%	3,4%	2,3%	3.00%	3,7%
***Students***	1,3%	0.00%	1,2%	0,9%	2,7%
***108. ORAL STATUS, OVERALL (clinical)***					
***All clinicians***	8,68%	2,89%	9,7%	8,36%	10,36%
***Dentists***	13,54%	6,9%	14,3%	12,8%	15,5%
***Students***	5,08%	0,9%	6,6%	5,2%	5,3%
***109. RADIOGRAPHIC INFORMATION***					
***All clinicians***	6,45%	12,72%	5,08%	3,38%	9,23%
***Dentists***	5,68%	8,6%	4,6%	4,3%	7,3%
***Students***	7,03%	14,8%	5,4%	2,8%	11,1%
***110. RESTORATIVE Item Status, Specific***					
***All clinicians***	16,19%	12,14%	12,01%	19,22%	18,02%
***Dentists***	13,68%	8,6%	7,4%	17.00%	16,4%
***Students***	18,05%	13,9%	15,1%	20,8%	19,6%
***111. TOOTH STATUS, Specific***					
***All clinicians***	17,06%	14,45%	16,4%	19,93%	15,09%
***Dentists***	15,28%	12,1%	17,1%	17.00%	12,8%
***Students***	18,38%	15,7%	15,9%	22.00%	17,3%
***112. CARIES***					
***All clinicians***	15,32%	9,83%	8,55%	19,57%	18,69%
***Dentists***	13,54%	5,2%	5,1%	17,4%	18,3%
***Students***	16,65%	12,2%	10,9%	21,1%	19,1%
***113. DIET***					
***All clinicians***	2,36%	0,00%	2,08%	3,2%	2,48%
***Dentists***	2,33%	0,00%	2,9%	3.00%	1,8%
***Students***	2,38%	0,00%	1,6%	3,4%	3,1%
***114. TOOTH EXTRACTION/ABSENCE***		0,00%			
***All clinicians***	3,1%	0,00%	2,08%	3,2%	5,18%
***Dentists***	3,35%	0,00%	1,1%	2,6%	6,8%
***Students***	2,92%	0,00%	2,7%	3,7%	3,6%
***115. ENDODONTIC TREATMENT AND MANAGEMENT***					
***All clinicians***	3,47%	1,16%	3,93%	2,85%	4,73%
***Dentists***	2,18%	1,7%	1,7%	1,7%	3,2%
***Students***	4,43%	0,9%	5,4%	3,7%	6,2%
	
***TOTAL For All Clinicans, Entire Consultation***	**100,0%**	**10,73%**	**26,86%**	**34,86%**	**27,54%**

Overall use of concepts across both types of clinicians increased as the visit progressed from the initial quarter to the third (10.7%, 26.8%, and 34.8%, respectively), and then decreased in the final quarter (27.5%). There was a very similar frequency of utilization of concepts dealing with *'Health Status, Overall' *when the entire visit was examined (5.82% for dentists and 5.84% for students; see Table 3). However, the bulk of the utilization of these concepts by dentists resided in the first quarter (22.4%) of their dental visit as opposed to students (16.5%). Use of these concepts diminished in the second quarter, became more apparent in the third, and disappeared in the fourth. Other concepts suggested differing patterns of use between students and dentists: *'Personal Habits/Features' *(different in the second and fourth quarters); *'Attendance To Dental Care' *(different across the overall visit and for every quarter); *'Oral Hygiene (Appearance)' *(different across the overall visit and for every quarter); *'Oral Status, Overall' *(different across the overall visit and for every quarter); *'Radiographic Information' *(different in the first quarter);* 'Restorative Item Status, Specific' *(different across the overall visit and in particular during the first and second quarters); and *'Caries' *(different for the first and second quarters).

## Discussion

Dentists and students (as groups) took about as long to complete their visits, to be ready to see the patient for the first time in the operatory area, spent about the same time with the patient, and invested similar time evaluating the patient's mouth. Furthermore, both groups of clinicians used about the same number of DTPs, had a similar range of DTPs in conducting their visits, and they each omitted the use of the same two DTPs (DTP10 and DTP11). All in all, experts and students had broad similarities in their diagnostic strategies - a finding that has been previously reported for medical clinicians [42,48,49]. Apparently, both groups quickly generate explanations or speculations for what could be wrong in a given clinical case, presumably based on non-analytic reference to past experience. It has been stated, however, that experienced clinicians outperform students because they work from a wider repository of past clinical experiences, with marked differences in the precise structure of thinking processes [42] that are not always directly related to a common core of biomedical knowledge [50]. Other researchers have characterized this straightforward access to better interpretation as 'encapsulated' knowledge [51,52], which does not necessarily rely on basic science propositions [53,54] but instead draws on identifying clinical significance faster and more accurately.

In the present study we found differences in DTP use between experts and students. We will first discuss what those differences were, and separately contrast their significance against the common perception that experts are better able to discriminate earlier and faster the significance of information [55,56].

The differences we found were telling in how the groups approach the processing of information. Dentists identified key pieces of clinical information through their more frequent use of DTP2 (*'Diagnostic interpretation of clinical information'*). Students, meanwhile, resorted to searching for clarifying pieces of information through DTP3 (*'Judgment of the need for further general or clarifying enquiry'*) more often to make sense of clinical information. By the third quarter, or halfway through the visit, students had continued to judge the need for further general or clarifying enquiry (DTP3) more often than dentists, suggesting that well into the second half of the visit students still had horizons of interpretation that demanded searches for clarifying information about the patient's signs or symptoms.

These open ended efforts must be distinguished from the three DTPs (DTP7, DTP8, and in particular DTP9) that are important to determine the route of the clinical encounter [57]. Even though the overall use of DTP9 (*'Routine enquiry'*) did not reach statistical significance when the four quarters of the visit were jointly examined, dentists were eliciting information on routine information (DTP9) in the final quarter. Students, meanwhile, had the bulk of the clinical history preferentially covered in earlier quarters of the visit (Table 2). Students were more adept at following a stricter roadmap for the unfolding visit, as also suggested elsewhere [58]. We found that the student's plan seemed to hinge on acquiring information according to a routine enquiry plan in which they first completed the medical and dental history. Seasoned clinicians on the other hand felt at ease to address or re-visit routine enquiries later in the visit. Perhaps this was a strategy to double check information or to gain time to think of the next step in the visit [59].

Do differences in DTP and concept utilization constitute the hallmark of expertise? (We must preface the discussion of this question with a reminder that we are using a clinical case emphasizing restorative needs, with no emergency or complex management issues.) The prevailing interpretation among scholars is that clinicians go about solving diagnostic problems using approaches that are in part mandated by the complexity posed by the case [52,59] but also seem to modulate strategies depending on the cognitive stage during the pursuit of a solution, and the perceived pay-off of choosing between differing approaches. It was common to find among our participants that provisional interpretations of the case gained credence or were replaced as the dental visit progressed. Many participants, in particular students, considered inherent to decisions in dental practice such revolving cycle of diagnoses and management interactions. In this way not all the information was instantly available but rather assembled in an incremental, sprouting fashion.

Clinical training has traditionally implied that a careful analysis of the relation between signs, symptoms and diagnoses is the hallmark of clinical expertise [60]. But the actual relationships that are established between clinical information and diagnoses with increased clinical experience are difficult to identify. Schmidt and Boshuizen [61,62] postulated that such relationships represent qualitatively different types of knowledge. These interrelationships are generally more restricted to a basic science focus in junior students; relationships are somewhat enriched with clinical interpretations and clinically relevant knowledge when applied by intermediate clinicians, such as senior students; and they are largely based on multi-linked knowledge that connects clinical interpretations with other domains of expertise when in use by a seasoned clinician. Such perspective has been adopted as an interpretative framework in the dental environment [11]. Similar developmental continua have been demonstrated for cardiology cases across multiple levels of medical expertise [56]. Our findings are generally aligned with such trends. We would urge caution, however, by pointing out that the pedagogical implications of this perspective of clinical decision-making should not be taken as a scenario in which diagnostic thinking always occurs in the same way. There are various reasons for such a caveat. First, Eva [60] divided the interpretations of diagnostic activity as *analytic *and *non-analytic *processing of information. In the first one, a careful evaluation of signs and symptoms and the possible associated diagnoses was conducted, and these linkages were explicitly scrutinized (e.g., '*Does that fractured back tooth hurt?*') or a sign elicited (e.g., vertical percussion of the fractured tooth). According to the second interpretation (non-analytic processing), clinicians may identify salient aspects of the signs and symptoms posed by a clinically meaningful amount of information. For example, in this study clinicians had to decide to restore or extract third molars: they looked decayed, were difficult to keep free of plaque, and while in reasonable occlusion positions, their value as teeth were lower than other teeth. If the path to attain a sufficient degree of certainty about the diagnosis is short and direct, a pattern is recognized and we can label it as 'non-analytical.' It is to be expected that the two approaches coexist in differing degrees and at diverse stages of the restorative dentistry decision-making effort [60] or they share some common characteristics [9] rather than constituting entirely dissimilar processing heuristics.

Secondly, an important question is how the pattern is acquired in a non-analytical approach. Regardless of whether such recognition occurs instantly or progressively, a decision leading to clinical action is made. A decision to intervene seemingly depends on one clinical decision-making feature being significant enough to justify the restoration on its own (e.g., evident signs of an advanced, active carious lesion), or an accumulation of features giving rise to the same effect. It has been established that a series of negative factors influences a clinician's decision making process such as when seeing a tooth with an old and 'ugly' filling, or when it presents marginal defects. These negative patient attributes can exert a cumulative influence on teeth being assigned for restoration more often than teeth with fewer negative factors [63,64].

Finally, case complexity also likely plays a role for different levels of clinician expertise in defining a case as either routine or emergency, or simple or difficult [9,37,38]. We strived to create a patient profile that primarily posed restorative challenges, as this was the objective of our research.

The absence of DTP10 and DTP11 was not unexpected, as we had reported the same feature in an unrelated group of dental students [9]. Although our standardized case may have had insidious shortcomings that stopped clinicians from identifying their own failure to make general or specific enquiries when reviewing the visit, it is possible this variation stemmed from differing complexities of the cases in our research and in the original model proposed by Gale and Marsden [38]. Regardless of how often these thoughts (DTP10 and DTP11) may come to clinicians in real life when the visit has ended (e.g., "*I forgot to ask about Z symptom...*"), we assume that any clinician who suddenly realizes he/she has forgotten to follow a line of enquiry would correct this immediately by asking about the matter. Such amendment(s) could not be categorized as DTP10 nor DTP11. Participants in the Gale and Marsden's study considered that cases were of similar complexity and typical of their usual performance [38]: a situation that resembled our own findings (data not reported). Absence of these DTPs may suggest greater difficulty in attaining a diagnosis, and thus give rise to retrospective self-assessments of performance. This explanation may also account for Gale and Marsden's [38] dismissal of 'non-analytical'pattern-recognition since their research involved highly-complex endocrinology and neurology tasks.

The use of concepts throughout the visit followed to a large extent the trends identified within the DTPs. An examination of Table 3 suggests there were differences between experts and students. Experts were more likely to ask early in the visit about general health status, and throughout most of the visit seemed to use more concepts related to dental visit patterns, oral hygiene, and overall dental status, than students. Conversely, students resorted more frequently to the use of radiographic, restorative, and dental caries concepts earlier on in the visit than dentists. It would not be unreasonable to suggest that students set out early on to identify clinical or radiographic signs of disease, whereas seasoned clinicians seemed to be more interested in investing their effort in establishing a clinical picture coupled to the patient's medical and dental history. While the latter, more relaxed approach may be characteristic of the initial appointment with a patient being seen for the first time, the contrast between the information content speaks to the differing priorities driving the strategies of the two groups of clinicians: Reyna et al. [55,56,65] pointed out that higher expertise is associated with relying on fewer dimensions of information before feeling comfortable at making clinical decisions, thus becoming more effective and parsimonious in such tasks.

The present body of information is derived from a fairly typical number of participants in qualitative research. The attention to detail to dissect the recorded visit and interview materials afforded a mosaic of cognitive strategies during the evaluation of clinical needs. We adhered to the theoretical approach used in Gale and Marsden's model by contrasting levels of expertise: seasoned dentists involved in private dental practice, compared with senior dental students in the last seven months of their clinical training. Students were able to provide comprehensive care and engage in relevant clinical decision-making efforts. A separate methodological consideration pertains to the fidelity enhancement of a real-life situation by using a 'live' clinical encounter: by removing the nuances that a live patient scenario can introduce into standardized observations we aimed to characterize human thinking. Because we used a secluded environment with a single patient who was trained to provide identical answers to similar questions, and to interact in a homogeneous way with every clinician, we feel that we procured as uninfluenced a setting as possible. However, a realistic interpretation of what the research setup offered was not an entirely unobtrusive clinical encounter. The remote possibility that interviewees are affected by their 'observed' status is acknowledged; our findings may be tainted by clinicians trying to 'excel' under test conditions, or trying to provide the information that the interviewer wants to hear. The only complete assurance against such potential biases is to undertake recordings of clinical encounters without informed consent - clearly an unethical design. Finally, the statistical analysis must be taken with reservations as qualitative research does not aim to render representative results. Therefore, the unadjusted alpha level may be a limitation of the study. This is always the case with non-experimental designs, and the lack of comparable studies made it unrealistic to undertake detailed power calculations.

In the present manuscript we have made no attempt to ascertain whether individual clinicians or groups of clinicians reached more appropriate diagnoses or treatment plans. Instead we subjectively ascertained that the management measures proposed fell within the limits of what was customary and reasonable for a clinical case. Interestingly, the considerable variation across individual clinicians in terms of how much time they took to evaluate the clinical case, did not have an influence on the time differences between the groups of students and experts. In terms of DTPs and concepts, differences did exist between students and dentists. Differences were not major, probably because we were very specifically focused on restorative treatment needs: associated decisions may resort more (or more frequently) to contrasts between idealized images of what appropriate restorations should look like [9-11] instead of a more analytic processing of information. The latter could be called upon if a more complex decision-making path demanded a wider/larger array of DTP or concepts in response to an unusual challenge (e.g., differential diagnosis of a case compatible with trismus, trigeminal neuralgia, and/or gross caries in a lower third molar), or a more complex challenge (e.g., differential diagnosis of mucosal changes vaguely suggestive of squamous cell carcinoma).

## Conclusions

To our knowledge, this is one of the few reports that examine in detail the developmental cognitive processes involved in dental decision-making using a 'real' live standardized patient. We recognized similarities and differences directly ascribable to two levels of decision-making experience/expertise by using a cognitive psychology model. We then identified concepts clinically relevant to restorative dentistry. The underlying benefit derived from using a single patient case is that stability and fidelity were enhanced as much as possible. Differences across the two groups were thus more likely to be developmental, which connote expert versus student heuristics, rather than case-specific differences derived from multiple clinical cases. Future work should address the strategies and concepts used to diagnose and ascertain treatment needs for presenting complaints, as opposed to 'routine' evaluation of restorative needs, as a likely example of how we may further tease out the use of analytic (deductive reasoning) and non-analytic (pattern recognition) approaches in dental clinical decision-making. By creating more clinical scenarios and testing them as cases to be solved by student and expert clinicians (and/or various levels of expertise represented by general practitioners and specialists) we should be able to expand the knowledge base in this area. Specifically, to ascertain the tolerance to risk and the perception of risk; the minimim sufficient profile of information that allows a clinical decision to be made; and the qualitative and quantitative differences in information processing that are specific to acute clinical care (e.g., acute odontalgia in a seemingly intact tooth) or to diagnostically complex scenarios (e.g., recurrent periodontal abscess in a poorly controlled diabetic patient), in contrast to the more 'routine' restorative care for patients of record. At a more general level, appraising the costs ascribable to various decision outcomes will help defend improved pedagogical and clinical guidelines.

## Abbreviations

DTP: diagnostic thinking processes. ICDAS: International Caries Detection & Assessment System criteria [39]

## Competing interests

The authors declare that they have no competing interests.

## Authors' contributions

GM led the design and analysis phases of the study, as well as steering the manuscript and associated presentations to completion/delivery. SS supported study design and acted as the main advisor in qualitative methods and stability assessment throughout the research; he had secondary responsibility for manuscript development. SM was in charge of data management, quantitative contrasts, and supported the various stages of manuscript development. LG provided major input to study design and undertaking, as well as assembling the contacts with expert clinicians; he had secondary responsibility for manuscript development. HE conducted most clinical arrangements, supported standardized patient recruitment and selection, conducted the screening of pool of patients, and assembled components of the clinical case; he had secondary responsibility for manuscript development. All authors read and approved the final manuscript.

## Authors' information

Not applicable

## Pre-publication history

The pre-publication history for this paper can be accessed here:

http://www.biomedcentral.com/1472-6831/10/11/prepub

## Supplementary Material

Additional file 1 **Appendix 1**. Identification of DTP and examples in transcripts. Click here for file

Additional file 2 **Appendix 2**. List of concepts identified and allocated to 98 categories in 15 groups of concepts. Click here for file

## References

[B1] Barrows HS Problem-Based Learning. An Approach to Medical Education 1980 Springer. New York, NY

[B2] Barrows HS The clinical reasoning of randomly selected physicians in general medical practice Clin Invest Med 1982 5 49 55 7116714

[B3] Elstein AS Shulman LS Sprafka SA Medical Problem Solving: An Analysis of Clinical Reasoning 1978 Cambridge, MA. Harvard University Press

[B4] Grembowski D Milgrom P Fiset L Dental decision-making and variation in dentist service rates Soc Sci Med 1991 32 3 287 94 10.1016/0277-9536(91)90106-M 2024138

[B5] Bader JD Shugars DA Agreement among dentists' recommendations for restorative treatment J Dent Res 1993 72 5 891 6 8501287 10.1177/00220345930720051001

[B6] Bader JD Shugars DA Variation in dentists' clinical decisions J Publ Health Dent 1995 55 3 181 8 10.1111/j.1752-7325.1995.tb02364.x 7562733

[B7] Brantley CF Bader JD Shugars DA Nesbit SP Does the cycle of rerestoration lead to larger restorations? J Am Dent Assoc 1995 126 1407 13 7594013 10.14219/jada.archive.1995.0052

[B8] Shugars DA Bader JD Practice parameters in dentistry: where do we stand? J Am Dent Assoc 1995 126 1134 43 7560571 10.14219/jada.archive.1995.0332

[B9] Maupome G Sheiham A Clinical decision-making in restorative dentistry. Content-analysis of diagnostic thinking processes and concurrent concepts used in an educational environment Eur J Dent Educ 2000 4 143 152 10.1034/j.1600-0579.2000.040401.x 11168479

[B10] Maupomé G Sheiham A Explanatory models in the interpretations of clinical features of dental patients within a university Dental Education setting Eur J Dent Educ 2002 6 1 2 8 10.1034/j.1600-0579.2002.060102.x 11872067

[B11] Crespo K Torres JE Recio ME Reasoning process characteristics in the diagnostic skills of beginner, competent, and expert dentists J Dent Educ 2004 68 12 1235 44 15576812

[B12] Merrett MCW Elderton RJ An in vitro study of restorative dental treatment decisions and dental caries Br Dent J 1984 157 128 33 10.1038/sj.bdj.4805448 6591943

[B13] Cleaton-Jones P Hargreaves JA Fatti LP Chandler HD Grossman ES Dental caries diagnosis calibration for clinical field surveys Caries Res 1989 23 195 9 10.1159/000261177 2736583

[B14] Elderton RJ Nuttall NM Variation among dentists in planning treatment Br Dent J 1983 154 201 6 10.1038/sj.bdj.4805041 6573898

[B15] ADEA Commission on Change & Innovation in Dental Education Hendricson WD Andrieu SC Chadwick DG Chmar JE Cole JR George MC Glickman GN Glover JF Goldberg JS Haden NK Meyerowitz C Neumann L Pyle M Tedesco LA Valachovic RW Weaver RG Winder RL Young SK Kalkwarf KL Educational strategies associated with development of problem-solving, critical thinking, and self-directed learning J Dent Educ 2006 70 9 925 936 16954414

[B16] Crawford JM Adami G Johnson BR Knight GW Knoernschild K Obrez A Patston PA Punwani I Zaki AM Licari FW Curriculum restructuring at a North American dental school: Rationale for change J Dent Educ 2007 71 4 524 531 17468314

[B17] Benner P Taking a stand on experiential learning and good practice Am J Crit Care 2001 10 60 62 11153186

[B18] Flack VF Atchison KA Hewlett ER White SC Relationships between clinician variability and radiographic guidelines J Dent Res 1996 75 2 775 82 10.1177/00220345960750020701 8655774

[B19] Espelid I Radiographic diagnoses and treatment decisions on approximal caries Community Dent Oral Epidemiol 1986 14 265 70 10.1111/j.1600-0528.1986.tb01069.x 3466745

[B20] Kay EJ Nuttall NM Relationship between dentists' treatment attitudes and restorative decisions made on the basis of simulated bitewing radiographs Community Dent Oral Epidemiol 1994 22 71 4 10.1111/j.1600-0528.1994.tb01575.x 8205782

[B21] Bader JD Shugars DA What do we know about how dentists make caries-related treatment decisions? Community Dent Oral Epidemiol 1997 25 97 103 10.1111/j.1600-0528.1997.tb00905.x 9088698

[B22] Barrows HS Clarke J, Leedham J Keynote Address: Problem-based Learning in Medicine Aspects of Educational Technology 10: Individualized Learning 1976 Philadelphia PA: Kogan Page

[B23] Reitman WR Shelley MW, Bryan GL Heuristic Decision Procedures, Open Constraints and the Structure of Ill- Defined Problems Human Judgements and Optimality 1964 John Hoboken NJ: Wiley and Sons

[B24] Hoffman PJ The paramorphic representation of clinical judgement Psychol Bull 1960 57 116 31 10.1037/h0047807 14402414

[B25] Ledley RS Lusted LB Reasoning foundations of medical diagnosis Science 1959 130 9 21 10.1126/science.130.3366.9 13668531

[B26] Hammond KR Summers DA Cognitive dependence on linear and non-linear cues Psychol Rev 1965 72 215 24 10.1037/h0021798 14324558

[B27] Wulff HR Rational Diagnosis and Treatment 1981 Oxford: Blackwell

[B28] Young MJ Eisenberg JM Williams SV Hershey JC Comparing aggregate estimates of derived thresholds for clinical decisions Health Serv Res 1986 20 6, Part I 763 80 3949540 PMC1068926

[B29] Berkey CS Douglass CW Valachovic RW Chauncey HH McNeil BJ Statistical methods for comparing dental diagnostic procedures Community Dent Oral Epidemiol 1990 18 169 76 10.1111/j.1600-0528.1990.tb00051.x 2201479

[B30] Douglass CW McNeil BJ Clinical decision analysis methods applied to diagnostic tests in dentistry J Dent Educ 1983 47 11 708 12 6581205

[B31] Mileman PA Vissers T Purdell-Lewis DJ The application of decision-making analysis to the diagnosis of approximal caries Comm Dent Health 1986 3 65 81 3457617

[B32] Maryniuk GA Anusavice KJ Clinical Decision-Making and Cost- Effectiveness: Impact on Treatment Choices Quality Evaluation of Dental Restorations. Criteria for Placement and Replacement 1989 Hanover Park IL: Quintessence Publishing Co. Inc

[B33] Weinstein MC Fineberg HV Clinical Decision Analysis 1980 Philadelphia PA: W.B. Saunders

[B34] Tulloch JFC Antczak AA Wilkes JW The application of decision analysis to evaluate the need for extraction of asymptomatic third molars J Oral Maxillofac Surg 1987 45 855 63 10.1016/0278-2391(87)90236-9 3309225

[B35] Feinstein AR Clinical Judgment 1967 Huntington NY: Krieger

[B36] Scadding JG Diagnosis: the clinician and the computer Lancet 1967 290 7521 877 882 10.1016/S0140-6736(67)92608-6 4168324

[B37] Norman GR Problem-solving skills, solving problems and problem-based learning Med Educ 1988 22 279 86 10.1111/j.1365-2923.1988.tb00754.x 3050382

[B38] Gale J Marsden P Medical Diagnosis. From Student to Clinician 1983 Oxford: Oxford University Press

[B39] Thyne JM The Psychology of Learning and Techniques of Teaching 1966 London: University of London Press

[B40] Page G Bordage G Allen T Developing key-feature problems and examinations to assess clinical decision-making skills Acad Med 1995 70 3 194 201 10.1097/00001888-199503000-00009 7873006

[B41] Gale J Marsden P Clinical problem solving; the beginning of the process Med Educ 1982 16 22 6 10.1111/j.1365-2923.1982.tb01214.x 7057720

[B42] Grant J Marsden P The structure of memorised knowledge in students and clinicians: an explanation for diagnostic expertise Med Educ 1987 21 92 8 10.1111/j.1365-2923.1987.tb00672.x 3574171

[B43] Criteria Manual, International Caries Detection and Assessment System (ICDAS): Baltimore, MD 03/12-14/05. NIDCR, ADA, IADR. Consulted 10/23/08 in http://www.dundee.ac.uk/dhsru/docs/ICDAS%20II%20criteria%20document%20September%2010.doc

[B44] Holsti OR Lindzey G, Aronson E Content Analysis Handbook of Social Psychology, Vol. 2, Research Methods 1968 Reading MA: Addison-Wesley

[B45] Weber RP Basic Content Analysis. SAGE University Papers, Series: Quantitative Applications in the Social Sciences 1985 Newbury Park, CA: Sage Publications Inc

[B46] Krippendorff K Content Analysis. An Introduction to its Methodology. The SAGE CommText Series 1980 5 Newbury Park, CA: Sage Publications Inc

[B47] Holsti OR Content Analysis for the Social Sciences and Humanities 1969 Reading MA: Addison-Wesley

[B48] Neufeld VR Norman GR Feightner JW Barrows HS Clinical problem solving by medical students: a cross-sectional and longitudinal analysis Med Educ 1981 15 315 322 10.1111/j.1365-2923.1981.tb02495.x 6973686

[B49] Bordage G Zacks R The structure of medical knowledge in the memories of medical students and general practitioners: categories and prototypes Med Educ 1984 18 406 416 10.1111/j.1365-2923.1984.tb01295.x 6503748

[B50] Grant J Marsden P Primary knowledge, medical education and consultant expertise Med Educ 1988 22 173 179 10.1111/j.1365-2923.1988.tb00002.x 3405111

[B51] Woods N Howey EHA Brooks LR Norman GR Speed kills? Speed, accuracy, encapsulations and causal understanding Med Educ 2006 40 973 979 10.1111/j.1365-2929.2006.02556.x 16987187

[B52] Verkoeijen PPJL Rikers RMJPm Schmidt HG Van De Wiel MWJ Kooman JP Case representation by medical experts, intermediates and novices for laboratory data presented with or without a clinical context Med Educ 2004 38 617 627 10.1046/j.1365-2923.2004.01797.x 15189258

[B53] Rikers RM Loyens SM Schmidt HG The role of encapsulated knowledge in clinical case representations of medical students and family doctors Med Educ 2004 38 1035 1043 10.1111/j.1365-2929.2004.01955.x 15461648

[B54] Rikers RM Loyens SM Winkel Te Schmidt HG Sins PH The role of biomedical knowledge in clinical case reasoning: A lexicon decision study Academic Med 2005 80 945 9 10.1097/00001888-200510000-00015 16186615

[B55] Reyna VF Lloyd FJ Brainerd CJ Schneider S, Shanteau J Memory, development, and rationality: An integrative theory of judgment and decision-making Emerging Perspectives on Judgment and Decision Research 2003 New York: Cambridge University Press

[B56] Reyna VF Lloyd FJ Physician decision making and cardiac risk: Effects of knowledge, risk perception, risk tolerance, and fuzzy processing J Exper Psych: Applied 2006 12 3 179 95 10.1037/1076-898X.12.3.179 16953744

[B57] Gale J Marsden P The role of the routine clinical history Med Educ 1984 18 96 100 10.1111/j.1365-2923.1984.tb00980.x 6700454

[B58] Chambers DW Geissberger M Toward a competency analysis of operative dentistry technique skills J Dent Educ 1997 61 10 795 803 9385322

[B59] Norman GR Eva KW Doggie diagnosis, diagnostic success and diagnostic reasoning strategies: an alternative view Med Educ 2003 37 676 677 10.1046/j.1365-2923.2003.01528.x 12895245

[B60] Eva KW What every teacher needs to know about clinical reasoning Med Educ 2004 39 98 106 10.1111/j.1365-2929.2004.01972.x 15612906

[B61] Schmidt HG Boshuizen HPA On the origin of intermediate effects in clinical case recall Mem Cognit 1993 21 338 351 8316096 10.3758/bf03208266

[B62] Schmidt HG Boshuizen HPA On acquiring expertise in medicine Educ Psychol Rev 1993 5 205 221 10.1007/BF01323044

[B63] Maupomé G A comparison of senior dental students and normative standards with regard to caries assessment and treatment decisions to restore occlusal surfaces of permanent teeth J Prosth Dent 1998 79 5 596 603 10.1016/S0022-3913(98)70183-0 9597615

[B64] Maupomé G Cumulative assessment of factors leading to restorative decisions in an educational environment. A graphical demonstration using an in vitro case Operative Dentistry 2000 25 4 336 43 11203840

[B65] Reyna VF Brainerd CJ Fuzzy-trace theory: An interim synthesis Learn Individ Diff 1995 7 1 75 10.1016/1041-6080(95)90031-4

