# Supplementary material for: Diagnostic thinking and information used in clinical decision-making: a qualitative study of expert and student dental clinicians
Source: BMC Oral Health. 2010 May 13;10:11. doi: 10.1186/1472-6831-10-11 (PMC2879228; doi:10.1186/1472-6831-10-11)
Supplement: Additional file 1 — Appendix 1. Identification of DTP and examples in transcripts. [file 1472-6831-10-11-S1.DOC]

**Appendix 1. Identification of DTP and examples in transcripts.**

DTP 1: Pre-diagnostic interpretation of clinical information. Clinician # 18, minute 14: (Upon first intra-oral examination of lower right posterior area, and asking about pain associated with fractured molar) “*Soft tissues and periodontal (…) look quite healthy*. *We’ll confirm against x-rays…*”.

DTP 2: Diagnostic interpretation of clinical information. Clinician # 14, minute 19: “*There are a couple of recession areas I want to make a note of… And just recession, buccal of 19, and 21 and 22. (…) I am glad it is not sensitive.*”

DTP 3: Judgment of need for further enquiry, not stemming from either pre-diagnostic or diagnostic interpretations. Clinician # 11, minute 14: (Upon first intra-oral examination) “*Have your wisdom teeth ever bothered you? No?*”

DTP 4: Expecting, searching for, or planning to search for specific features of disease or treatment of disease. Clinician # 6, minute 19: (After reviewing radiographs) “*Are you having any kind of pain, trouble, anything like that…?*”

DTP 5: Reinterpretation of clinical information, when no new information has been added. Clinician # 1, minute 33: (Re-examining the fractured tooth) “*I’m just worried about the nerve on that tooth… But since it hasn’t hurt, and there is nothing on the x-ray, I’d guess it can be safe to just rebuild the crown.*”

DTP 6: Reinterpretation of clinical information arising from the addition of new information. Clinician # 22, minute 8: (After ascertaining presenting complaint) “*Has (the temporary filling) worked, have you had any pain…?*”

DTP 7: Enquiry responsive to elicited information. Clinician # 13, minute 22: “*So you have had some over-the-counter filling material on the tooth, and it’s been there for three-four months – right? No problems?*”

DTP 8: Enquiry determined by the clinician's interpretation. Clinician # 12, minute 18: (After the intra-oral examination, and upon re-evaluation of the radiographs) “*When did you say that you placed that temporary? Three months ago?*”

DTP 9: Routine enquiry. Clinician # 22, minute 8: “*What about your medical history? Have you had any heart, lung, kidney problems…? Nothing? I read in your questionnaire that you had some gall bladder surgery… Have you had any complications? You’re not allergic to anything, are you?*”

DTP 12: Active confirmation of an interpretation. Clinician # 1, minute 38: “*I think most of these cavities are not your fault. You have taken good care of your teeth. These are cavities in pit and fissures, or developmental flaws. I do not think it is neglect. Even if you nibble on sugars…*”

DTP 13: Active elimination of an interpretation. Clinician # 8, minute 32: “*If I saw any problems with your third molars, aside from just those cavities that you have on them, I might recommend having them extracted. But I really don’t see anything but the cavities. (…) Let us keep an eye on them. If they end up being a problem in the future, we might consider having a third molar extraction. At this time, I really don’t see it – you have space for them.*”

DTP 14: Postponement of either confirmation or elimination of a possible interpretation. Clinician # 14, minute 24: “*OK, the ‘Canyon tooth’* (tooth 31)*: Question mark, endo. Post and core, crown? (…) In order to save that tooth, we’ll need a crown. There are some options;* (even though) *it is not bothering you, it may still have some decay, but we’ll decide about root canal treatment when we establish how much* (decay) *there is…*”
