# Supplementary material for: Diagnostic thinking and information used in clinical decision-making: a qualitative study of expert and student dental clinicians
Source: BMC Oral Health. 2010 May 13;10:11. doi: 10.1186/1472-6831-10-11 (PMC2879228; doi:10.1186/1472-6831-10-11)
Supplement: Additional file 2 — Appendix 2. List of concepts identified and allocated to 98 categories in 15 groups of concepts. [file 1472-6831-10-11-S2.DOC]

**Appendix 2. List of concepts identified and allocated to 98 categories in
15 groups of concepts.**

**101. HEALTH STATUS, OVERALL**

overall status

systemic or chronic illness, personal

systemic or chronic illness, family

infectious disease

previous experience with anesthetics

medication, past and present

allergies

**102. PERSONAL HABITS/FEATURES.**

smoking

alcohol intake

coffee intake

illegal drugs

employment

**103. PHYSICAL STATUS, OVERALL.**

weight

fitness

**104. ATTENDANCE (to dental care).**

periodic attendance as a patient

change of practitioner

affordability of dental care

perceived need (by the patient) of dental care

previous bad experience with dental care-dentist

**105. COMPLAINT/SIGN/SYMPTOM.**

color change in teeth

pain in the lower third of the face, non-dental origin

pain in the lower third of the face, dental origin (now)

pain in the lower third of the face, dental origin (recently)

positive appraisal of dental sign upon probing or visualization

generalized dental sensitivity

**106. ORAL HYGIENE (actions related to).**

tooth brushing, frequency

tooth brushing, technique

non-fluoride mouth rinses, dental floss and/or detection tablets

patient's exposure to fluoride, administered either by public health or individual measures

**107. ORAL HYGIENE (appearance).**

as related to periodontal structures

as related to caries

presence of tartar, debris and/or plaque

disseminated tooth stains (extrinsic to tooth structure)

**108. ORAL STATUS, OVERALL (actual clinical appearance).**

disseminated tooth stains (intrinsic to tooth structure)

abrasion/attrition signs

features of occlusion in the patient

signs of periodontal/gingival disease (in mucosa)

signs of periodontal/gingival disease (in alveolar bone)

presence of periodontal pockets

presence of intra-oral pathologic process (non-perio origin)

presence of extra-oral pathologic process (non-perio origin)

previous dental history of disease/abnormalities (orthodontics), measures implemented to correct them and their outcome

**109. RADIOGRAPHIC INFORMATION.**

presence of periapical pathologic processes

presence of abnormal periodontal processes

presence of carious lesion(s)

**110. RESTORATIVE ITEM STATUS, SPECIFIC (actual clinical appearance).**

presence of marginal defects

surface corrosion

"large" restoration and/or multiple restorations

food and plaque-retention points

restoration fracture(s) (non-marginal) or proximal overhangs

time interval that restorative items have been in the mouth

incorrect design of cavity preparation and/or need to re- design it according to new requirements, arising from a new assessment of, or new developments in, the case

"old", "ugly" or "faulty" restorations

lack or inadequacy of anatomic features in restoration

Temporary/provisional restoration

Crown (metal, PFM, other)

**111. TOOTH STATUS, SPECIFIC (actual clinical appearance).**

fracture(s)

"demineralized" area(s)

"sticky" fissure(s) or defect(s) in enamel or restoration

circumscribed pigmentation

presence of "loose" substance upon probing, whether of dental or non-dental origin

circumscribed area with darker shade as compared to remaining enamel in the tooth

pit and fissure sealants

**112. DENTAL CARIES.**

new lesion (actual presence in situ)

residual lesion (actual presence in situ)

recurrent lesion (actual presence in situ)

undefined lesion (actual presence in situ)

new lesion (possible presence in situ)

residual lesion (possible presence in situ)

recurrent lesion (possible presence in situ)

undefined lesion (possible presence in situ)

previous caries experience

caries as a result of use of systemic medication – xerostomia/Salivary Gland Hypofunction

caries as a result of sugars in diet

caries as a result of socio-economic deprivation

caries as a result of socio-cultural factors

caries as a result of extreme individual susceptibility

caries as a result of not being a regular patient

caries as a result of inadequate oral hygiene

caries as a result of inadequate preventive measures (personal)

caries as a result of inadequate preventive measures (public)

**113. DIET.**

adequate intake and variety of foodstuffs, compatible with a sufficiently-balanced nutrition appropriate for good health

highly-industrialized, additive-added food

"junk-food" intake

"natural food" intake

sugary snack and beverage intake, total intake

cariogenic sweeteners in food/beverages, total intake

sugary snack and beverage intake, intake frequency

cariogenic sweeteners in food/beverages, intake frequency

**114. TOOTH EXTRACTION/ABSENCE**

Third molars

Third molar extraction, non-surgical

Third molar extraction, surgical

Congenitally missing teeth

**115. ENDODONTIC TREATMENT AND MANAGEMENT**

Percussion or pressure on teeth

Root canal treatment

Tooth/teeth sensitive to temperature changes (hot/cold)

Foul smell in mouth

Swelling, localized in mouth or face
